# Weight status in individuals with autism spectrum disorder: A study protocol for systematic review and meta-analysis

**DOI:** 10.1097/MD.0000000000017274

**Published:** 2019-09-20

**Authors:** Xue-Ni Xie, Yong-Jiang Li, Xue Lei, Ya-Min Li

**Affiliations:** a Shuda College, Hunan Normal University; b Department of Pharmacy, The Second Xiangya Hospital of Central South University, Changsha, Hunan, China; c School of Psychology, University of Queensland, St Lucia, QLD, Australia; d Clinical Nursing Teaching and Research Section, The Second Xiangya Hospital of Central South University, Changsha, Hunan, China.

**Keywords:** autism spectrum disorder, obesity, overweight, prevalence, underweight

## Abstract

**Background:**

Weight status and autism spectrum disorder (ASD) are rising public health concerns. An increasing number of reports indicate that individuals with ASD may have unhealthy weight status, but the evidence is mixed. To understand the weight status in individuals with ASD and provide strategies for prevention and intervention, we describe the protocol for a systematic review and meta-analysis aimed at assessing the prevalence of obesity, overweight, and underweight in ASD.

**Methods:**

A broad range of key bibliographic databases including MEDLINE (PubMed), Embase, Cochrane, and ISI Web of Science will be searched to identify studies reporting the prevalence of obesity, overweight, and underweight in patients with ASD. Retrieved records will be independently screened by 2 authors and relevant estimates will be extracted from studies reporting data on obesity, overweight, and underweight prevalence among individuals with ASD. The assessment of study quality will be conducted primarily using the Newcastle–Ottawa scale and checklist proposed by the Joanna Briggs Institute. Prevalence estimates of obesity and overweight will be separately pooled using random-effects model. The pooled estimates will be summarized and presented by regional groupings. Subgroup analysis will be conducted for variables (such as study setting, participants’ age, and geographical region) across studies, depending on data availability. Between-study heterogeneity will be assessed using the *I*^2^ statistic and explored through subgroup analyses. This systematic review and meta-analysis will be reported following the preferred reporting items for systematic reviews and meta-analyses checklist and the meta-analysis of observational studies in epidemiology statements guidelines for meta-analysis and systematic reviews of observational studies.

**Results:**

In this study, we will outline details of the aims and methods on the meta-analysis of weight status of individuals with ASD.

**Conclusion:**

The results of this study will summarize the current data of weight status of individuals with ASD.

**Registration:**

PROSPERO-National Institute of Health Research (NIHR) Prospective Register of Systematic Reviews (CRD42019130790).

## Abstract 2

Supplemental Digital Content is available in the text

## 1 Introduction

Autism spectrum disorder (ASD) is one of the most common neurodevelopmental disorders. ASD usually appears in children by the age of 3 and is characterized by deficits in social interactions and communications and repetitive sensory-motor behaviors as well as stereotypic patterns of behaviors.^[1]^ ASD is a major pediatric health issue. According to the report from autism and developmental disabilities monitoring network, up to 3% of children in the US have ASD.^[2]^ ASD is associated with a range of high costs and families with autistic children are facing crushing economic burdens.^[3]^

Obesity and overweight are major public health issues, and the prevalence has been rising.^[4,5]^ Obesity and overweight are the leading cause of death and are considered as one of the major causes of morbidity such as diabetes, cardiovascular disease, and cancer.^[6–8]^ Obesity and overweight in children can cause early death.^[9]^ Childhood obesity also presents an increased risk of adult obesity and is associated with health risks.^[10,11]^ Obesity-related medical costs will rise to as high as $48 billion to $66 billion per year in the US and £1·9 to 2 billion per year in the UK by 2030.^[12]^ While the prevalence of underweight has not been rising,^[13]^ underweight among children and adolescents is associated with higher risk of various diseases and burdens of underweight is increasingly high in low- and middle-income countries.^[14]^

A growing number of studies have investigated the association between ASD and unhealthy weight status in children/adolescents and adults,^[15–17]^ while quiet variable results have been reported.^[18–20]^ Unlike underweight, the putative association between ASD and obesity/overweight might seem reasonable because, other than a disease, obesity, and overweight are often terms used to describe fatness and unhealthy dietary habit and lack of physical activities are recognized as the main drivers.^[21]^ It has been reported that individuals with ASD were less likely to participate in physical activities^[22]^ and ASD patients often have significantly dysregulated diet composition, with atypical selectivity (prefer more calorically dense foods).^[23]^ However, study results have not been consistent and to what extent are obesity and ASD are associated is not clear. More recently, high frequency of underweight class in children/adolescents with ASD has also been noted. Besides, the role of potential confounders in explaining the association has not been understood. Given the public health concerns of unhealthy weight status and ASD and the uncertainty about the association, it is of public health priority to assess the possible link and to evaluate the role of confounding factors on the relationship to design evidence-based prevention strategies.

The objective of this protocol is to conduct a systematic review and meta-analysis to estimate the global prevalence of obesity, overweight, and underweight in ASD based on existing literature. We specifically focus on whether, and to what extent, the prevalence of obesity, overweight, and underweight are significantly higher in individuals with ASD compared to those without. Also, possible confounding factors would be addressed by performing additional meta-analysis depending on data availability.

## 2 Methods

Methods for performing this systematic review and meta-analysis have been defined in advance following recommendations from meta-analysis of observational studies in epidemiology statements^[24]^ and the preferred reporting items for systematic reviews and meta-analyses (PRISMA).^[25]^

### 2.1 Selection criteria

#### 2.1.1 Study setting and population

All original, peer-reviewed studies that reporting data or estimates will be considered. We exclude case studies, reviews, meta-analysis, and meeting abstracts. Besides, to collect data as many as possible and summarize the global prevalence of obesity, overweight, and underweight in ASD, the control group setting is not a mandatory criterion for study inclusion. The representative populations will include children and/or adults with ASD. Study sample derived from the following sources will be considered:

(1) the general population;
(2) patients’ registers and databases;
(3) screening programs;
(4) clinical settings.

No geographical limitations will be applied when selecting studies.

#### 2.1.2 Definitions and outcome measures

The definition of ASD will include:

(1) categorical diagnosis according to the diagnostic and statistical manual of mental disorders or international classification of diseases;
(2) a validated ASD rating scales;
(3) records of medical system or registries of previous diagnosis of ASD;
(4) an affirmative answer from parents to the question “Did the doctor ever inform you that your kid has ASD?” about their children or from adults to the same question about themselves.

The definition of obesity/overweight will include:

(1) diagnosis report based on any of the internationally accepted body mass index cut-offs (Centers for Disease Control and Prevention,^[26]^ International Obesity Task Force,^[27]^ World Health Organization ^[28]^);
(2) records based on parental-report or self-report of obesity or overweight or directly measured data (height and weight).

### 2.2 Search strategy

A series of complementary search method will be applied for the strategy. Relevant studies will be identified through searching the electronic databases: Pubmed, Embase, Cochrane Library, and ISI Web of Science. The search strategy was first developed in Pubmed using Mesh subject headings combined with keywords around the 2 search components (obesity, overweight and underweight and ASD). Then, the manual search will be conducted through reviewing reference lists or citations follow-up of identified eligible articles and relevant articles. No language limitation will be applied. Detailed search strategy for electronic databases is available in online supplementary Appendix 1.

### 2.3 Identification and selection of studies

The records retrieved from electronic searches and manual searches will be grouped together and independently screened by 2 researchers for eligibility. Full-text copies would be obtained for records that potentially meet the eligibility. Then, these full-text articles will be independently assessed by 2 researchers for consideration of inclusion. Any disagreement about the eligibility will be resolved through consensus between the 2 researchers or discussion and a third author as arbitrator.

### 2.4 Data extraction

The data in included studies will be independently extracted by 2 researchers. The following data will be extracted for all included studies using a standardized form: publication information (author, published year); study information (country where the study conducted, study setting, data source, study period); general population information (sample size, age, and gender distribution); specific population information (ASD criteria, treatment or medication use, comorbidities); outcome information (obesity, overweight and underweight definition, point prevalence rates of obesity, overweight and underweight for overall sample or for specific subgroups); confounding factors (if available). If studies did not report such point prevalence but provided the number of obese participants or height and weight information in ASD population, the data will be collected and calculated to obtain the prevalence rates. Any discrepancies for the data extraction will be resolved through consensus between the 2 authors or discussion with the third author as arbitrator.

### 2.5 Study quality assessment

Two authors will independently assess the quality and bias in the included studies. Since there is no consensus on rating methods and appropriateness of quality assessment in systematic review and meta-analyses of observational studies. We will firstly use the Newcastle–Ottawa scale^[29]^ as recommended by the Cochrane collaboration, and then use the appraisal checklist proposed by the Joanna Briggs Institute,^[30]^ which is developed for studies reporting prevalence estimates and has been used in systematic review and meta-analysis. The results of 2 different rating systems will be referenced when performing sensitivity analysis and subgroup analysis. Any discrepancy in the rating of study quality and bias will be resolved by consensus between the 2 authors or through discussion with the third author as arbitrator.

### 2.6 Data analysis

We will first describe the characteristics of included studies in narrative text and baseline tables, and then perform meta-analysis to pool the prevalence of obesity/overweight in individuals with ASD. If available, meta-analysis of both crude and adjusted prevalence estimates will be conducted. Considering the variability between included studies, obesity/overweight prevalence pooled estimates and their 95% confidence intervals will be computed applying random-effects model of DerSimonian-Laird. Heterogeneity between studies will be assessed using the Cochran *Q* statistic and the *I*^2^ statistic.^[31]^*I*^2^ values of 25%, 50%, and 75% would be generally interpreted as low, medium and high heterogeneity, respectively. The presence of publication bias will be assessed by visually observing the funnel plot and by quantitative Egger^[32]^ and Begg^[33]^ tests. If publication bias is detected, the Duval Tweedie method will be used to obtain the adjusted estimates.^[34]^ Subgroup analysis and meta-regressions, depending on the data feasibility, would be performed considering the following covariates: study setting, gender, age, sample size, study period, geographical region, the method to assess ASD, the definition of obesity, overweight and underweight, comorbidities, medication use and quality of study. Also, sensitivity analysis will be performed to test the robustness of the pooled estimates. Statistical analyses will be performed in combination with Review Manager and Stata statistical software.

### 2.7 Presenting and reporting of results

**Figure 1:**
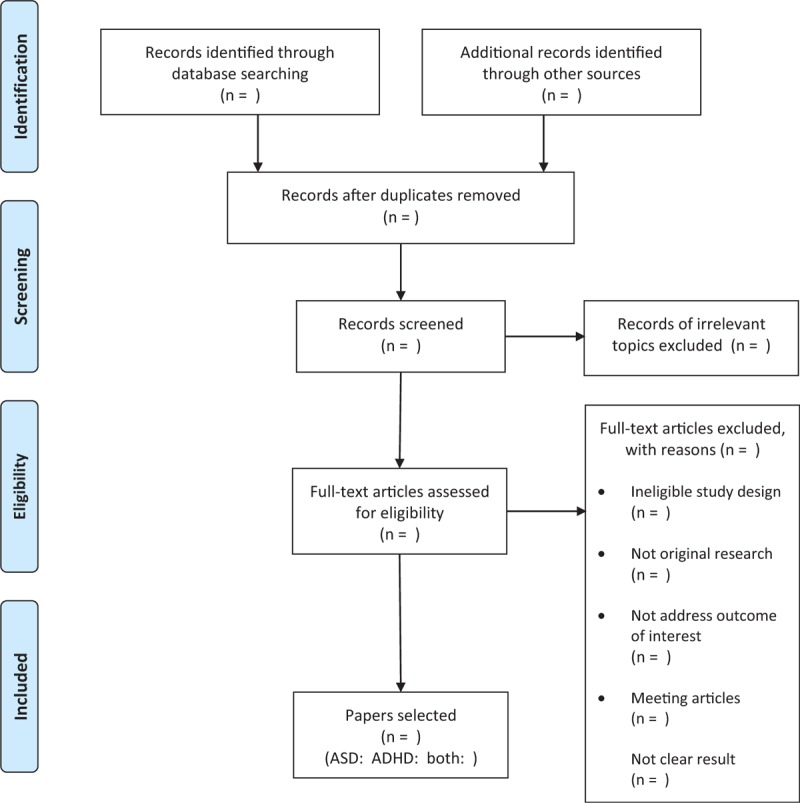
Flow chart of the study selection process.

The study selection process will be presented in a PRISMA flow chart (Fig. 1), and reasons for exclusion of studies will be provided. Data extracted from included studies will be summarized in baseline tables. The results of assessment of quality of study will be presented in tables showing scores in each domain. Raw data will be presented in baseline tables of individual studies, pooled estimates will be presented in forest plots and summary tables. Prevalence will be examined by subgroup analysis of variables described in Data analysis and presented in plots or tables where appropriate.

## Author contributions

**Conceptualization:** Ya-Min Li

**Data curation:** Xue-Ni Xie, Xue Lei

**Formal analysis:** Xue-Ni Xie, Yong-Jiang Li

**Funding acquisition:** Ya-Min Li

**Investigation:** Xue-Ni Xie, Xue Lei

**Methodology:** Yong-Jiang Li, Ya-Min Li

**Project administration:** Yong-Jiang Li

**Resources:** Xue-Ni Xie, Xue Lei

**Software:** Xue-Ni Xie, Xue Lei

**Supervision:** Yong-Jiang Li

**Validation:** Yong-Jiang Li

**Visualization:** Yong-Jiang Li

**Writing – original draft:** Xue-Ni Xie

**Writing – review & editing:** Xue-Ni Xie, Yong-Jiang Li, Xue Lei, Ya-Min Li

## Supplementary Material

Supplemental Digital Content

## References

[1] American Psychiatric Association. Diagnostic and statistical manual of mental disorders (DSM-5®). New York: American Psychiatric Pub; 2013.

[2] Christensen DL Maenner MJ Bilder D Prevalence and characteristics of autism spectrum disorder among children aged 4 years - early autism and developmental disabilities monitoring network, seven sites, United States, 2010, 2012, and 2014. Morbidity and mortality weekly report. Surveillance Summaries 2019;68:1–9. 10.15585/mmwr.ss6802a1 PMC6476327 30973853

[3] Rogge N Janssen J The economic costs of autism spectrum disorder: a literature review. J Autism Dev Disord 2019;49:2873–900. 30976961 10.1007/s10803-019-04014-z

[4] Garrido-Miguel M Cavero-Redondo I Alvarez-Bueno C Prevalence and trends of overweight and obesity in European children from 1999 to 2016: a systematic review and meta-analysis. JAMA Pediatr 2019 e192430 doi: 10.1001/jamapediatrics.2019.2430. 31381031 10.1001/jamapediatrics.2019.2430 PMC6686782

[5] Hales CM Fryar CD Carroll MD Trends in obesity and severe obesity prevalence in US youth and adults by sex and age, 2007-2008 to 2015-2016. JAMA 2018;319:1723–5. 29570750 10.1001/jama.2018.3060 PMC5876828

[6] Lee DH Keum N Hu FB Predicted lean body mass, fat mass, and all cause and cause specific mortality in men: prospective US cohort study. BMJ 2018;362:k2575. 29970408 10.1136/bmj.k2575 PMC6028901

[7] Twig G Yaniv G Levine H Body-mass index in 2.3 million adolescents and cardiovascular death in adulthood. N Engl J Med 2016;374:2430–40. 27074389 10.1056/NEJMoa1503840

[8] Lee DH Keum N Hu FB Comparison of the association of predicted fat mass, body mass index, and other obesity indicators with type 2 diabetes risk: two large prospective studies in US men and women. Eur J Epidemiol 2018;33:1113–23. 30117031 10.1007/s10654-018-0433-5

[9] Franks PW Hanson RL Knowler WC Childhood obesity, other cardiovascular risk factors, and premature death. N Engl J Med 2010;362:485–93. 20147714 10.1056/NEJMoa0904130 PMC2958822

[10] Simmonds M Llewellyn A Owen CG Predicting adult obesity from childhood obesity: a systematic review and meta-analysis. Obes Rev 2016;17:95–107. 26696565 10.1111/obr.12334

[11] Llewellyn A Simmonds M Owen CG Childhood obesity as a predictor of morbidity in adulthood: a systematic review and meta-analysis. Obes Rev 2016;17:56–67. 26440472 10.1111/obr.12316

[12] Wang YC McPherson K Marsh T Health and economic burden of the projected obesity trends in the USA and the UK. Lancet 2011;378:815–25. 21872750 10.1016/S0140-6736(11)60814-3

[13] Ezzati M Bentham J Di Cesare M Worldwide trends in body-mass index, underweight, overweight, and obesity from 1975 to 2016: a pooled analysis of 2416 population-based measurement studies in 128.9 million children, adolescents, and adults. Lancet 2017;390:2627–42. 29029897 10.1016/S0140-6736(17)32129-3 PMC5735219

[14] Jaacks LM Slining MM Popkin BM Recent trends in the prevalence of under- and overweight among adolescent girls in low- and middle-income countries. Pediatr Obes 2015;10:428–35. 25558987 10.1111/ijpo.12000 PMC4492920

[15] Dhaliwal KK Orsso CE Richard C Risk factors for unhealthy weight gain and obesity among children with autism spectrum disorder. Int J Mol Sci 2019;20:E3285. 31277383 10.3390/ijms20133285 PMC6650879

[16] Bicer AH Alsaffar AA Body mass index, dietary intake and feeding problems of Turkish children with autism spectrum disorder (ASD). Res Dev Disabil 2013;34:3978–87. 24029808 10.1016/j.ridd.2013.08.024

[17] Granich J Lin A Hunt A Obesity and associated factors in youth with an autism spectrum disorder. Autism 2016;20:916–26. 26893400 10.1177/1362361315616345

[18] Mari-Bauset S Llopis-Gonzalez A Zazpe-Garcia I Nutritional status of children with autism spectrum disorders (ASDs): a case-control study. J Autism Dev Disord 2015;45:203–12. 25194628 10.1007/s10803-014-2205-8

[19] Castro K Faccioli LS Baronio D Feeding behavior and dietary intake of male children and adolescents with autism spectrum disorder: a case-control study. Int J Dev Neurosci 2016;53:68–74. 27432261 10.1016/j.ijdevneu.2016.07.003

[20] Liu X Liu J Xiong X Correlation between nutrition and symptoms: nutritional survey of children with autism spectrum disorder in Chongqing, China. Nutrients 2016;8:E294. 27187463 10.3390/nu8050294 PMC4882707

[21] Prentice AM The emerging epidemic of obesity in developing countries. Int J Epidemiol 2006;35:93–9. 16326822 10.1093/ije/dyi272

[22] Must A Phillips S Curtin C Barriers to physical activity in children with autism spectrum disorders: relationship to physical activity and screen time. J Phys Act Health 2015;12:529–34. 25920014 10.1123/jpah.2013-0271 PMC4490003

[23] Evans EW Must A Anderson SE Dietary patterns and body mass index in children with autism and typically developing children. Res Autism Spectr Disord 2012;6:399–405. 22936951 10.1016/j.rasd.2011.06.014 PMC3427936

[24] Stroup DF Berlin JA Morton SC Meta-analysis of observational studies in epidemiology: a proposal for reporting. Meta-analysis of observational studies in epidemiology (MOOSE) group. JAMA 2000;283:2008–12. 10789670 10.1001/jama.283.15.2008

[25] Shamseer L Moher D Clarke M Preferred reporting items for systematic review and meta-analysis protocols (PRISMA-P) 2015: elaboration and explanation. BMJ 2015;350:g7647. 25555855 10.1136/bmj.g7647

[26] Kuczmarski RJ Ogden CL Guo SS 2000 CDC growth charts for the United States: methods and development. Vital Health Stat 11 2002;246:1–90. 12043359

[27] Cole TJ Bellizzi MC Flegal KM Establishing a standard definition for child overweight and obesity worldwide: international survey. BMJ 2000;320:1240–3. 10797032 10.1136/bmj.320.7244.1240 PMC27365

[28] de Onis M Onyango AW Borghi E Development of a WHO growth reference for school-aged children and adolescents. Bull World Health Organ 2007;85:660–7. 18026621 10.2471/BLT.07.043497 PMC2636412

[29] Wells GA Shea B O’Connell D The Newcastle-Ottawa Scale (NOS) for assessing the quality of nonrandomised studies in meta-analyses. http://www.ohri.ca/programs/clinical_epidemiology/oxford.asp Accessed September 7, 2019.

[30] Munn Z Moola S Lisy K Methodological guidance for systematic reviews of observational epidemiological studies reporting prevalence and cumulative incidence data. Int J Evid Based Healthc 2015;13:147–53. 26317388 10.1097/XEB.0000000000000054

[31] Higgins JP Thompson SG Quantifying heterogeneity in a meta-analysis. Stat Med 2002;21:1539–58. 12111919 10.1002/sim.1186

[32] Egger M Davey Smith G Schneider M Bias in meta-analysis detected by a simple, graphical test. BMJ 1997;315:629–34. 9310563 10.1136/bmj.315.7109.629 PMC2127453

[33] Begg CB Mazumdar M Operating characteristics of a rank correlation test for publication bias. Biometrics 1994;50:1088–101. 7786990

[34] Duval S Tweedie R Trim and fill: a simple funnel-plot–based method of testing and adjusting for publication bias in meta-analysis. Biometrics 2000;56:455–63. 10877304 10.1111/j.0006-341x.2000.00455.x

